# Dynamic-Step-Size Regulation in Pulse-Coupled Neural Networks

**DOI:** 10.3390/e27060597

**Published:** 2025-06-03

**Authors:** Jiayi Geng, Fanqing Ji, Shouliang Li, Yulin Shen, Zhen Yang

**Affiliations:** 1 School of Information Science and Engineering, Lanzhou University, Lanzhou 730000, China; gengjy2024@lzu.edu.cn (J.G.); jifq2020@lzu.edu.cn (F.J.); 2 Gansu Computing Center, Lanzhou 730030, China; shenyl@cc.gs.cn

**Keywords:** PCNN, image segmentation, random step, entropy

## Abstract

Pulse-coupled neural networks (PCNNs) are capable of segmenting digital images in a multistage unsupervised fashion; however, optimal output selection remains challenging. To address the above problem, this paper emphasizes the role of the step size, which influences the decreasing speed of the membrane potential and the dynamic threshold profoundly. A dynamic-step-size mechanism is proposed, utilizing trigonometric functions to adaptively control segmentation granularity, along with the supervised optimization of a single parameter ϕ via intersection over union (IoU) maximization, reducing tuning complexity. Thus, the number of groups of image segmentation becomes controllable and the model itself becomes more adaptive than ever for various scenarios. Experimental results further demonstrate the enhanced robustness under noise (92.1% Dice at σ=0.2), outperforming SPCNN and PCNN with IoU = 0.8863, Dice = 0.901, and 0.8684 s/image.

## 1. Introduction

Image segmentation remains a fundamental challenge in computer vision, where traditional PCNN models exhibit notable advantages in unsupervised segmentation due to their bio-inspired characteristics. However, their reliance on fixed step-size (ST) settings severely limits practical applications. It was Eckhorn’s research on the visual cortex neurons of cats that gave birth to the PCNN model. The PCNN model’s structure has multiple inputs and a single output and its neurons can work in both excitatory and inhibitory states. The parallel inputs are internally superimposed in time and space dimensions to form a nonlinear output. This phenomenon is the so-called coupling. The PCNN inherits those biologic characteristics and enables image pixels in neighboring areas or that have similar gray levels to cluster together.

During the past few decades, plenty of valuable research studies about this model have emerged in the image processing field. In 2007, Ma et al. [1] applied the time matrix of release pulse in image enhancement for the first time. In 2009, Zhan et al. [2] introduced a Spiking Cortical Model (SCM) which had lower computational complexity and higher accuracy than before. Two years later, Chen et al. [3] modified the sigmoid function of the SCM with a classical firing condition to further simplify the computational complexity. Recently, researchers have put more attention on the intrinsic activity mechanism of neurons, e.g., the heterogeneous PCNN [4,5] and the non-integer-step model [6] are becoming new focuses in the image processing field. In 2021, Liu et al. [7] proposed the continuous-coupled neural network (CCNN), which could generate a variety of stochastic responses stimulated by different driven signals [8,9]. Besides the above research, Wang et al. [10] proposed an infrared–visible image fusion method using the snake visual mechanism and a PCNN. It mimicked snake vision and enhanced fusion quality, broadening PCNNs’ applications in image fusion. In recent years, PCNNs have found numerous applications in the field of image processing. For example, Hu et al. proposed a remote sensing image reconstruction method based on a parameter-adaptive dual-channel pulse-coupled neural network (Dual-PCNN) in Ref. [11]. This method achieved excellent results in image noise reduction and fusion, further expanding the application scope of PCNNs in practical image processing. PCNNs have great potential for developing image segmentation algorithms, whose performance deeply relies on appropriate parameter selection. However, how to select the right outputs is still an issue, although many adaptive parameter filtering methods have been proposed [2,12,13,14,15,16,17].

Although prior studies [1,2,3] have advanced parameter adaptation strategies, the dynamic adjustment mechanism for the step size remains underexplored. Traditional models (e.g., SPCNN) face three key limitations:

- Uncontrollable granularity: a large ST leads to over-segmentation (noise sensitivity), while a small ST causes under-segmentation (detail loss).
- Generalization constraints: a fixed ST struggles to adapt to diverse gray distributions and complex scenes.
- Parameter tuning complexity: a manual ST adjustment is required to balance accuracy and efficiency.

This work aimed to address these issues through dynamic-step-size adaptation.

Here, we try to explore the mechanism of step size working on a dendritic tree. The rest of this paper is organized as follows: Section 2 begins with a brief inspection of a basic PCNN model and its pulse generator mechanism. Section 3 reviews two very significant related works, the Simplified PCNN (SPCNN) and non-integer-step-index model. Section 4 is devoted to the study of the step size in model operation, and some experiments are performed. Section 5 concludes the whole work.

## 2. Neuron Structure

**Figure 1:**
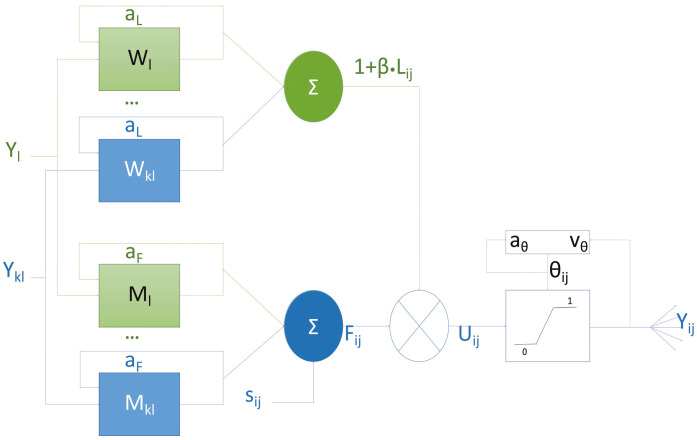
Model of PCNN’s neuron structure.

The past decades have witnessed great achievements in the neuron structure modification and parameter setting of PCNNs. As a type of bionic neural network [18], there are four main components in a neuron of a PCNN: the dendrite, the membrane potential, the dynamic threshold, and the action potential, which were also redefined by Lindblad and Kinser [19] as the feeding input, the linking input, the internal activity, and the pulse generator, respectively. The fundamental elements of Eckhorn’s model consist of distinct leaky integrators which can be implemented by first-order recursive digital filters [20]. The neuron exhibits bidirectional connectivity, allowing for both the reception of information from neighboring neurons and the transmission of signals to subsequent ones. As shown in Figure 1, the feeding synapses, *F*, receive an external stimulus that is the main input signal, whereas the linking synapses, *L*, receive auxiliary signals to modulate the feeding inputs. The nonlinear connection modulation component, also known as the internal activity of the neuron, *U*, consists of a linear linking, a feedback input, and a bias. During the process of signal transmission, neuronal electrical signal intensity is full, so two decay factors $a_{F}$ and $a_{L}$ are set in the transfer process from feeding synapses to other neurons and linking synapses to other neurons, respectively. When a neuron receives a postsynaptic action potential from neighboring neurons, it charges immediately and then decays exponentially. Each neuron is denoted with indices (indices (i,j)), and one of its neighboring neurons is denoted with indices (k,l). The mathematical expressions of a PCNN are as follows:

(1) $$L_{ij}\left[n\right]=e^{-a_{L}}L_{ij}\left[n-1\right]+V_{L}\sum_{kl}W_{ij,kl}Y_{kl}\left[n-1\right]$$

(2) $$F_{ij}\left[n\right]=e^{-a_{F}}F_{ij}\left[n-1\right]+V_{F}\sum_{kl}M_{ij,kl}Y_{kl}\left[n-1\right]$$

(3) $$U_{ij}\left[n\right]=F_{ij}\left[n\right]\left(1+\beta L_{ij}\left[n\right]\right)$$

(4) $$\theta_{ij}\left[n\right]=e^{-a_{\theta }}\left[n-1\right]+V_{\theta }Y_{ij}\left[n\right]$$

And the pulse generator is

(5) $$Y_{ij}\left[n\right]=\left\{\begin{array}{cc} 1, & U_{ij}\left[n\right]>\theta_{ij}\left[n\right]\\ 0, & else. \end{array}\right.\;$$

- $L_{ij}\left[n\right]$: linking input term, where $a_{L}$ is the linking decay factor, $V_{L}$ is the linking amplification coefficient, and $W_{ij,kl}$ denotes the neighborhood weighting matrix;
- $F_{ij}\left[n\right]$: feeding input term, with $a_{F}$ as the feeding decay factor, $V_{F}$ the feeding amplification coefficient, and $M_{ij,kl}$ the spatial coupling matrix;
- $U_{ij}\left[n\right]$: internal activity, modulated by β (linking strength coefficient);
- $\theta_{ij}\left[n\right]$: dynamic threshold, governed by $a_{\theta }$ (threshold decay factor) and $V_{\theta }$ (threshold amplification coefficient);
- $Y_{ij}\left[n\right]$: pulse output (one indicates firing, zero otherwise).

**Figure 2:**
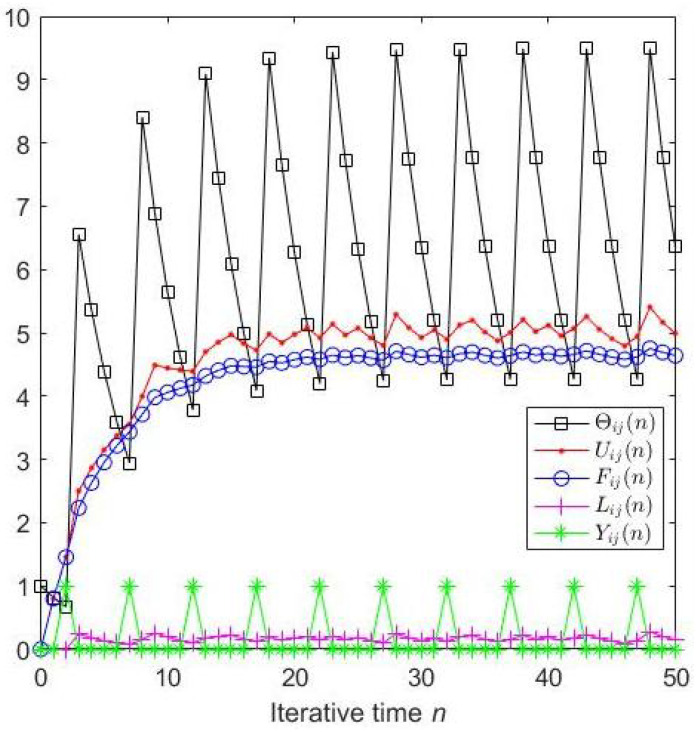
Tracking the parameter of a specific neuron.

A particular neuron tends to fire at a certain frequency if all the parameters are determined; as illustrated in Figure 2, after three iterations, the neuron prefers firing periodically. In fact, all neurons show the aforementioned behavior when perceiving different external stimuli, which means the information can be fully perceived merely after a finite number of iterations.

## 3. Related Works

Recent advances in visual perception modeling [21] demonstrate that PCNN-based approaches significantly outperform traditional methods in complex background segmentation. Combined with adaptive parameter strategies [22] and comprehensive application reviews [23], these developments highlight the growing potential of PCNN in real-time vision systems.

In addition to related works such as the Simplified PCNN (SPCNN) and the non-integer-step-index PCNN mentioned above, there are also studies that explore the PCNN model in depth from different dimensions. Reference [24] conducted research on the non-coupled PCNN. Based on the traditional non-coupled PCNN model, a linking term was introduced to improve it. By analyzing the firing mechanism of the improved model, it was found that its firing time and interval changed with the firing states generated by the neighborhood and its own firing conditions in each iteration process. That study also delved into the influence of parameters such as the linking weight matrix and the linking coefficient on the network output characteristics, revealing that under specific parameter settings, the non-coupled PCNN could exhibit the network output characteristic of image edge detection, which was verified through numerous experiments. That research achievement enriched the research system of the PCNN model and provided important references for subsequent studies.

Moreover, Xu et al. [25] proposed a novel adaptively optimized PCNN model for hyperspectral image sharpening. They designed a SAM-CC strategy to assign hyperspectral bands to the multispectral bands and proposed an improved PCNN considering the differences in neighboring neurons, which was applied to remote sensing image fusion and achieved good results, expanding the application scenarios of PCNNs in remote sensing image processing. In addition to these studies, Qi et al. proposed an adaptive dual-channel PCNN model [26]. They applied it to infrared and visible image fusion, combined with a novel image decomposition strategy, and obtained excellent results, which further broadened the application scope of PCNNs in image fusion .

This section gives a brief overview of SPCNN and non-integer-step-index PCNN, proposed by Chen [3]. The former put forward a smart automatic parameter setting method; the latter emphasized the leverage of the step size for neurons’ perceptibility.

### 3.1. SPCNN and Adaptive Parameter

Compared with previous version, the SPCNN not only has a more concise model expression but also makes great progress in automatic parameter setting. The internal activity of the SPCNN consists of a leak integrator, linking input, and feeding input.

(6) $$U_{ij}\left[n\right]=S_{ij}\left(1+\beta V_{L}\sum_{kl}W_{ij,kl}Y_{kl}\left[n-1\right]\right)+e^{-a_{F}}U_{ij}\left[n-1\right],$$

where $S_{ij}$ is the external stimulus, and other parameters have the same meaning as indicated above.

The dynamic threshold $E_{ij}$ is rewritten as

(7) $$E_{ij}\left[n\right]=e^{-a_{E}}E_{ij}\left[n-1\right]+V_{E}Y_{ij}\left[n-1\right],$$

where $V_{E}$ and $e^{-a_{E}}$ have the same meaning as $V_{\theta }$ and $e^{-a_{\Theta }}$ in Equation (4). For clarity, these symbols are used in this paper.

In addition, the pulse generator of the SPCNN is inherited from the PCNN without any changes. Several main parameters are calculated as follows:

(8) $$a_{F}=log\left(\frac{1}{\sigma }\right),$$

(9) $$\beta =\frac{S_{max}/S^{\prime }}{6V_{L}},$$

(10) $$V_{E}=a^{-a_{F}}+1+6\beta V_{L},$$

(11) $$V_{L}=1,$$

(12) $$a_{E}=log\left(\frac{V_{E}}{S_{max}\left(1-e^{-3a_{F}}/\left(1-e^{-a_{F}}+6\beta V_{L}e^{-a_{F}}\right)\right)}\right),$$

where σ denotes the standard deviation of the normalized intensities of an original image. More detail on the proof process can be found in Chen’s paper [3].

### 3.2. Non-Integer-Step-Index PCNN

The fact is that the neurons of the PCNN model are usually based on non-integer time, which is often ignored in the discrete form. Thus, non-integer-step-index PCNN changes the integer step into a decimal one to achieve preferable balance between resolution and computational complexity.

To handle a non-integer δt in discrete implementations, we adopted a linear interpolation between adjacent iterations. For δt=n+α (n∈Z,0<α<1), the membrane potential is updated as

$$U_{ij}\left[t+\delta t\right]=\left(1-\alpha \right)U_{ij}\left[t+n\right]+\alpha U_{ij}\left[t+n+1\right]$$

This ensures smooth transitions while avoiding subscript indexing issues.

(13) $$U_{ij}\left[t+\delta t\right]=S_{ij}\left(1+\beta V_{L}\sum_{kl}W_{ij,kl}Y_{kl}\left[t\right]\right)+e^{-a_{F}\delta t}U_{ij}\left[t\right]$$

(14) $$E_{ij}\left[t+\delta t\right]=e^{-a_{E}\delta t}E_{ij}\left[t\right]+V_{E}Y_{ij}\left[t\right]$$

(15) $$Y_{ij}\left[t+\delta t\right]=\left\{\begin{array}{cc} 1, & U_{ij}\left[t+\delta t\right]>E_{ij}\left[t\right]\\ 0, & else \end{array}\right.\;$$

where δ is the step size.

Though the idea is enlightening, in fact, it is not easy to realize the model in this form. People are used to storing ht output of every stage in an array or matrix, but decimals cannot correspond to a subscript of an array. Instead, the step size can change at each iteration step.

## 4. Research on Step Size

Generally speaking, in the study of artificial neural networks, it is difficult to monitor the internal processes. If the results of a model are not satisfactory, the first reaction is often to modify parameters. In fact, the model may work well, except that the output does not meet human expectations. If more subgraphs are split, will the result be more accurate? Or can the output include more detail if fewer subgraphs are split? From Equation (13), we obtain

(16) $$U_{ij}\left[t\right]=S_{ij}\left(1+\beta V_{L}\sum_{kl}W_{ij,kl}Y_{kl}\left[t-\delta t\right]\right)+e^{-a_{F}\delta t}U_{ij}\left[t-\delta t\right],$$

Subtracting Equation (16) from Equation (13) yields

(17) $$\begin{array}{cc} U_{ij}\left[t+\delta t\right] & =S_{ij}\beta V_{L}\left(\sum_{kl}W_{ij,kl}Y_{kl}\left[t\right]-\sum_{kl}W_{ij,kl}Y_{kl}\left[t-\delta t\right]\right)\\ \; & +\left(1+e^{-a_{F}\delta t}\right)U_{ij}\left[t\right]-e^{-a_{F}\delta t}U_{ij}\left[t-\delta t\right] \end{array}$$

Regarding *t* as n−δt, one gets

(18) $$\begin{array}{c} U_{ij}\left[n\right]=S_{ij}\beta V_{L}(\sum_{kl}W_{ij,kl}Y_{kl}\left[n-\delta t\right]-\sum_{kl}W_{ij,kl}Y_{kl}\\ \left[n-\delta t]\right)+\left(1+e^{-a_{F}\delta t}\right)U_{ij}\left[n-\delta t\right]-e^{-a_{F}\delta t}U_{ij}\left[n-2\delta t\right] \end{array}$$

Expanding Equation (13) using a first-order Taylor series approximation for $e^{-a_{F}\delta t}$, we have:

(19) $$e^{-a_{F}\delta t}\approx 1-a_{F}\delta t+\frac{{\left(a_{F}\delta t\right)}^{2}}{2}-\ldots$$

Neglecting higher-order terms ($O(\delta t^{2})$), we substitute the expression into Equation (13) and derive the discrete form as shown in Equation (17). This approximation ensures computational tractability while maintaining the dynamic coupling behavior.

Equation (18) indicates that a variable ST dynamically adjusts segmentation sensitivity by modulating two factors:

- The historical decay rate of membrane potential ($e^{-a_{F}ST}$);
- The neighborhood pulse coupling difference ($\sum Y_{kl}\left[n-1\right]-\sum Y_{kl}\left[n-2\right]$).

Compared to a fixed ST, a variable ST enables adaptive granularity control across iterations.

Equation (18) bridges non-integer- and variable-step-size models. By substituting δt=ST and allowing ST to vary per iteration, we extend the discrete PCNN framework to support dynamic-step adaptation. This formulation preserves the biological coupling mechanism while enabling the adaptive control of the membrane potential decay ($e^{-a_{F}ST}$) and neighborhood pulse coupling.

Assuming δt=1 in Equation (13), we get Equation (6), and we get Equation (18) from Equation (13), so if we assume δt=1 in Equation (18), then we get a *U* equal to one in Equation (6):

(20) $$\begin{array}{cc} U_{ij}\left[n\right] & =S_{ij}\beta V_{L}\left(\sum_{kl}W_{ij,kl}Y_{kl}\left[n-1\right]-\sum_{kl}W_{ij,kl}Y_{kl}\left[n-2\right]\right)\\ \; & +\left(1+e^{-a_{F}\delta t}\right)U_{ij}\left[n-1\right]-e^{-a_{F}\delta t}U_{ij}\left[n-2\right] \end{array}$$

For programming convenience, the indices should be integers. Thus, if δt changes, we have to use a new step size (ST) to replace δt, and the former equation is rewritten as

(21) $$\begin{array}{cc} U_{ij}\left[n\right] & =S_{ij}\beta V_{L}\left(\sum_{kl}W_{ij,kl}Y_{kl}\left[n-1\right]-\sum_{kl}W_{ij,kl}Y_{kl}\left[n-2\right]\right)\\ \; & +\left(1+e^{-a_{F}ST}\right)U_{ij}\left[n-1\right]-e^{-a_{F}ST}U_{ij}\left[n-2\right] \end{array}$$

Equation (16) equals

(22) $$U_{ij}\left[n-1\right]=S_{ij}\left(1+\beta V_{L}\sum_{kl}W_{ij,kl}Y_{kl}\left[n-2\right]\right)+e^{-a_{F}ST}U_{ij}\left[n-2\right]$$

as t=n−1. Let us use *n* instead of *n+1* and introduce $U_{ij}\left[n\right]$ into Equation (22); we can get the same result:

(23) $$U_{ij}\left[n\right]=S_{ij}\left(1+\beta V_{L}\sum_{kl}W_{ij,kl}Y_{kl}\left[n-1\right]\right)+e^{-a_{F}ST}U_{ij}\left[n-1\right].$$

Via the former derivations, we extend Equation (16), which is an important hidden intermediate procedure that shows that the ST can actually change across iterations. The ST can affect the image segmentation result significantly. When ST equals to one, the model is the traditional SPCNN, and it becomes the non-integer-step-index model when ST is decimal. However, the latter is still a fixed-step-size model, whose application scope is limited.

**Figure 3:**
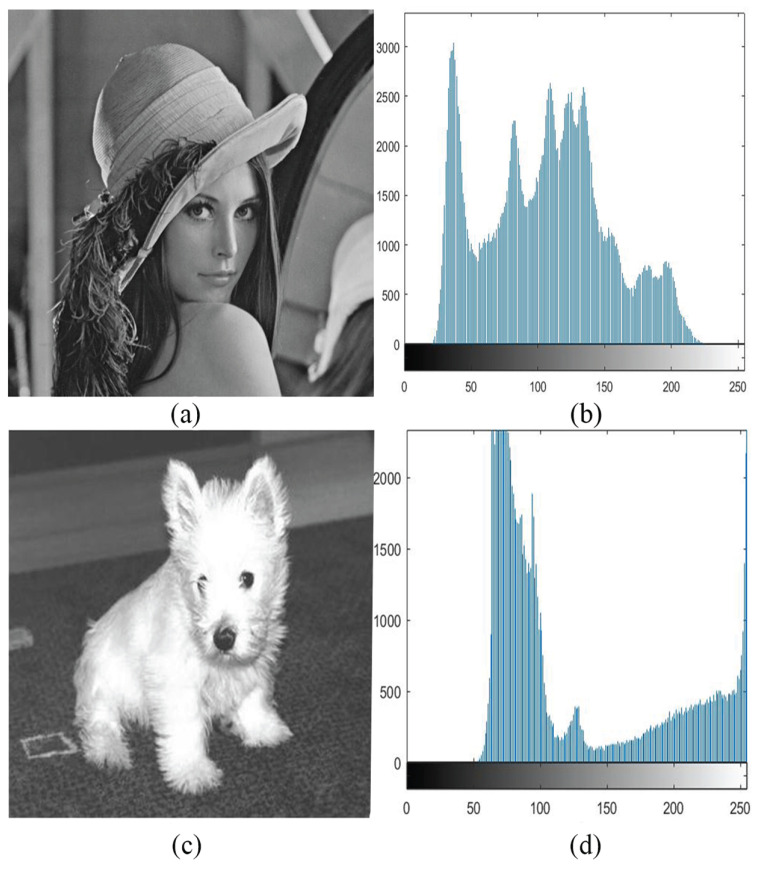
(**a**) “Lena”. (**b**) Frequency statistics for each gray value of Lena. (**c**) “Puppy”. (**d**) Frequency statistics for each gray value of Puppy.

The PCNN can capture both grayscale level and position information of pixels in an image. Here, we explore the function of the ST in grayscale perception. Figure 3a,c show two images used in the experiment, and Figure 3b,d are their corresponding histograms. We took “Lena” as an example to show how the ST works on determining a threshold according to grayscale level and position.

**Figure 4:**
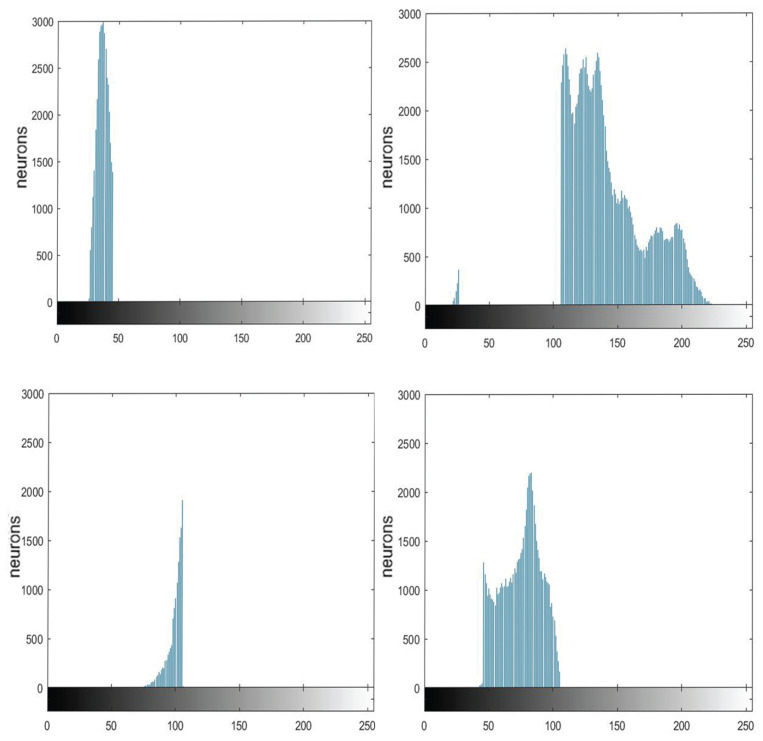
SPCNN divides the image into four parts.

**Figure 5:**
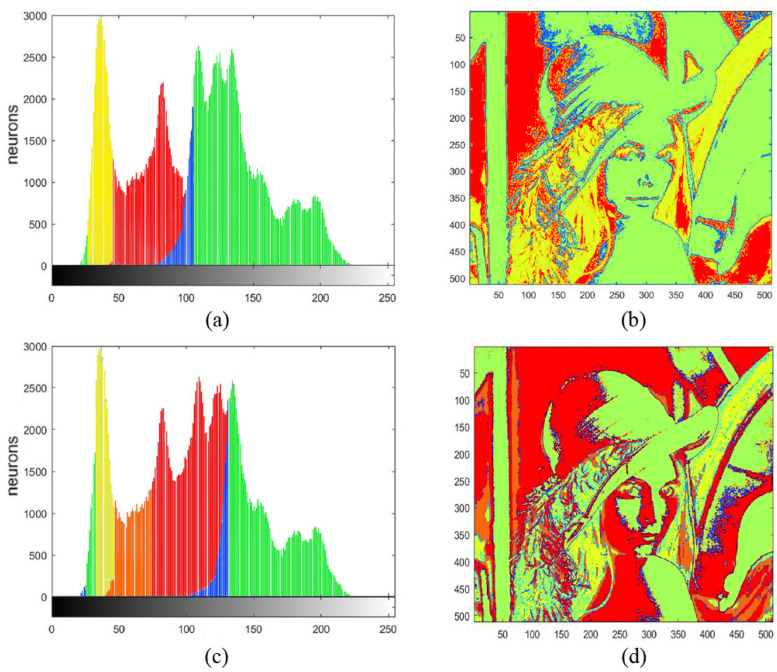
(**a**) Segmentation of Lena when ST = 1; (**b**) pixel map of Lena with the same colors as in the last picture; (**c**) Segmentation of Lena when ST = 0.6, (**d**) pixel map of Lena when ST = 0.6.

Figure 4 displays the histograms of four components of the image “Lena” separated by the SPCNN with ST = 1. For simplicity, these four histograms were marked with different colors and the corresponding pixels were labeled with the same color as in Figure 5a and b, respectively. In addition, Figure 5c,d represent the marked histograms and image when ST = 0.6. The pixels were recorded and displayed with the same colors as in Figure 5b,d. When the ST became smaller, the largest interval (e.g., the blue part in Figure 5c) tended to split first. In the meanwhile, these adjacent intervals occupied the extreme parts of the plot. Notice that there was always a small blue area between the two largest intervals, which was less affected. In fact, that area represented the boundary between the foreground and the background. We could use it as the threshold for binary segmentation. It can be observed that the segmentation result did not strictly rely on the threshold, as the spatial information between different pixels was taken into account, i.e., a neuron was easier to trigger if its neighbors had already fired, since the convolution operation could include the neighboring neurons’ information. This phenomenon is known as synchronous firing, which enables the PCNN to remove isolated noise. This ability becomes weak for a smaller ST, which causes more clusters to emerge, as shown in Figure 5a,c. Thus, the neurons at the edge of two groups can even cluster into a new group, like the orange part in Figure 5c. On the contrary, we can obtain more complete and continuous results when the ST is increased.

However, the selection of a suitable ST is full of challenges since a larger value enables more neurons to synchronously fire but a lower ability to distinguish objects, while a smaller ST has a higher distinctive ability, but more noise emerges. As the SPCNN segmentation deeply rely on the grayscale information of the image, we could let the images with similar grayscale values share the same step size. We can determine the best step size of these images with manually segmented results using an evaluation metric like the intersection over union (IoU).

**Figure 6:**
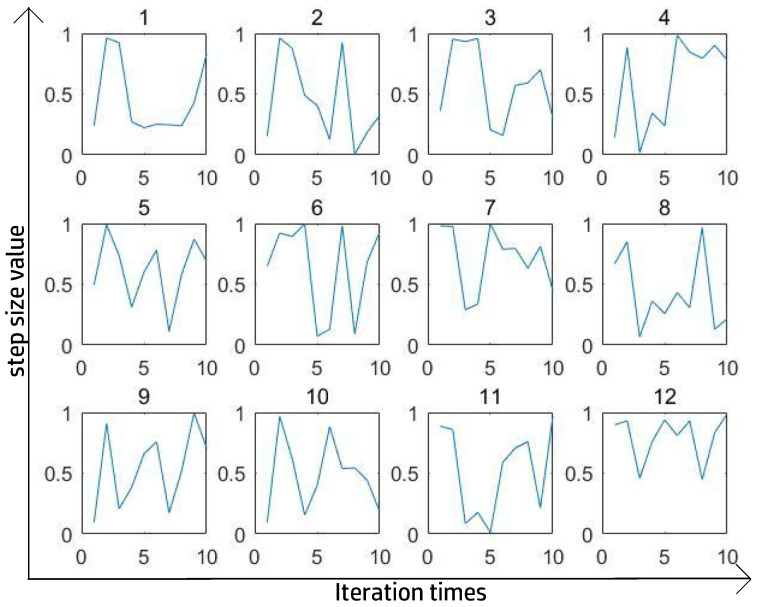
Best ST in SPCNN’s supervised experiment on Lena.

**Figure 7:**
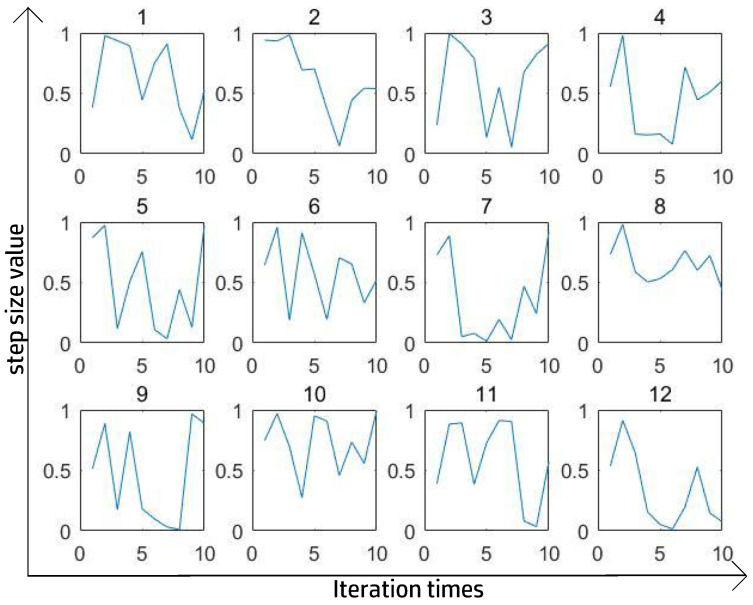
Best ST in SPCNN’s supervised experiment on Puppy.

Figure 6 and Figure 7 show the best 12 ST curves in 100 iterations with a totally random ST at each step. The criterion for perfect segmentation was to ensure the highest IoU. After a large number of experiments and after removing some extreme values, we found that the trigonometric function could fit the ST curve relatively well and consumed less time than using a totally random number.

The ST value was expected to be in the interval [0, 1] to ensure the PCNN model can sufficiently distinguish between inputs, so we assumed ST to be

(24) ST=0.5sin(wt+ϕ)+0.5

where *t* is the iteration time, and ϕ is a randomness parameter affecting the time of the first peak of ST curve.

**Figure 8:**
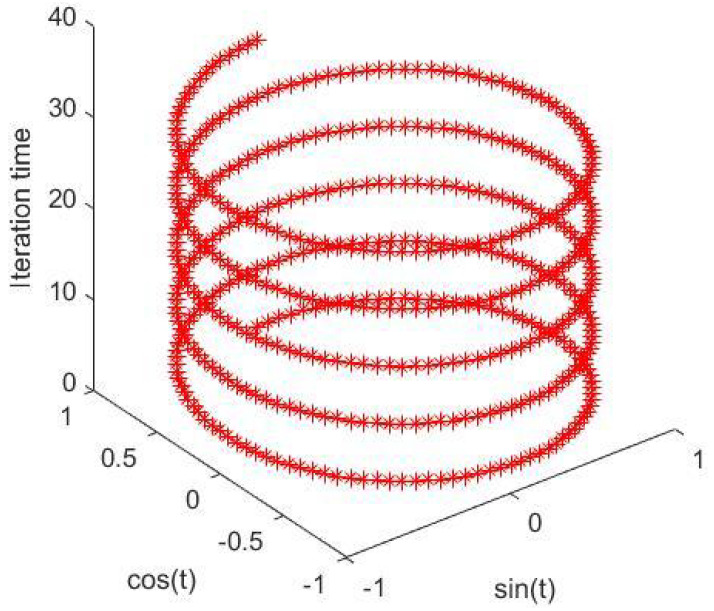
Conceptual representation of how the model take steps.

The sinusoidal function (Equation (23)) was chosen over linear/exponential alternatives for three reasons: (1) Periodicity ensures cyclic exploration of granularity levels, avoiding local minima; (2) The bounded output [0,1] matches the ST range; (3) Parameter efficiency (only ϕ and *w*). Biologically, the sine function mimics neural oscillations observed in cortical networks [8], where rhythmic firing enhances feature discrimination. Mathematically, the derivative d(ST)/dt=0.5wcos(wt+ϕ) naturally modulates edge sensitivity by amplifying gradient changes (see Figure 8).

According to (22), an ST of $U_{ij}\left[n-2\right]$ is one step behind, so it is equivalent to the cosine operation. During the iteration process, the sine item of $U_{ij}\left[n-1\right]$ and the cosine item of $U_{ij}\left[n-2\right]$ work on $U_{ij}\left[n\right]$ together. Therefore, the internal activity *U* varies spirally, which is a unique characteristic of this new mechanism.

Extensive experiments showed that *w* was related to the standard deviation of the normalized image. Thus, let *w* be equal to

(25) $$w=log(\frac{1}{\sigma })$$

and ϕ is in the range [0, pi/*w*].

**Figure 9:**
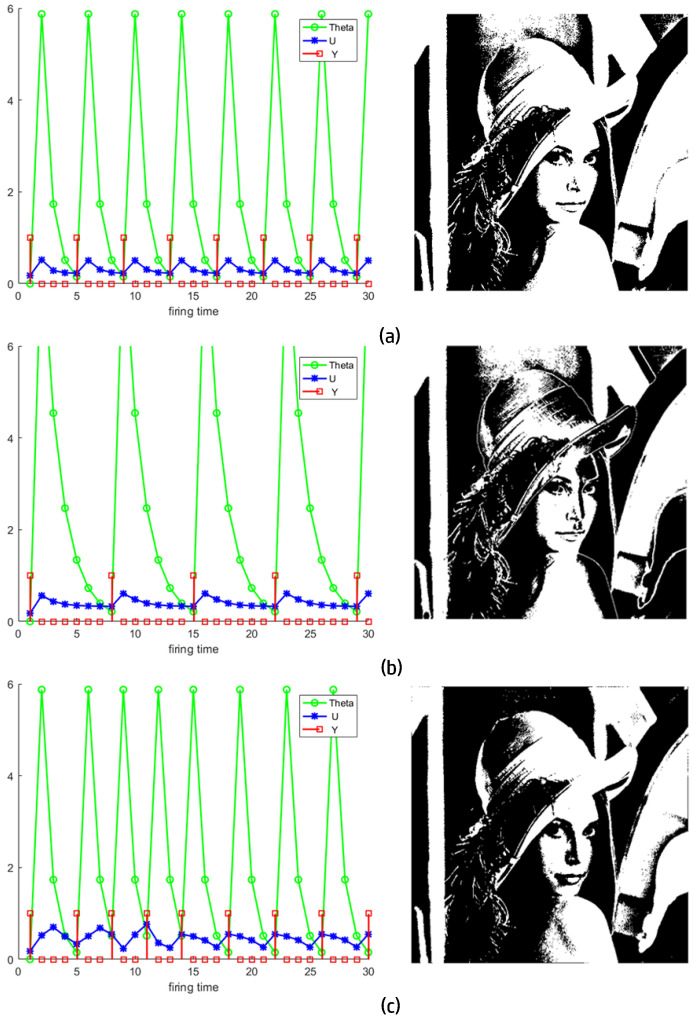
The first column records the changes in the parameter, and the second column shows the effect on the corresponding image segmentation (**a**) ST = 1, (**b**) ST = 0.5, (**c**) ST = 0.5∗sin(1.5681n)+0.5.

Figure 9 shows the neurons activity of the aforementioned scheme and the final segmentation results (Algorithm 1). When the ST gets smaller, the image tends to be divided into more parts, but for the variable-step-size PCNN, it is an exception. It is striking that although the ST in Equation (24) is always smaller than one, Figure 9c has less parts than Figure 9a. In this example, ϕ was not considered.

However, it is clear that in the first iteration of the variable-step-size PCNN in Figure 9c, the model narrows down the target area and ignores those bright parts of the wall behind the character, compared to what SPCNN achieves in Figure 9a.

To determine the best ST, as *w* is related to the statistical information of the image, we only considered the value of ϕ to simplify the problem. Because Figure 9c showed better results than Figure 9a, we believed that the ST in the former outperformed the latter, and the ϕ of the former was recorded until we encountered a better ST. Many ways are available to find the best segmentation; one of the most effective ones is via the IoU metric.

**Figure 10:**
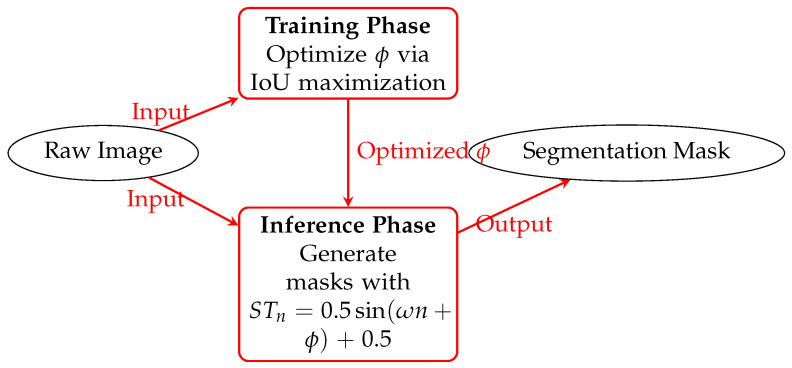
Dynamic-step-size PCNN framework. Training phase (**top**) optimizes ϕ; inference phase (**bottom**) applies adaptive $ST_{n}$. Arrows indicate data flow.

**Figure 11:**
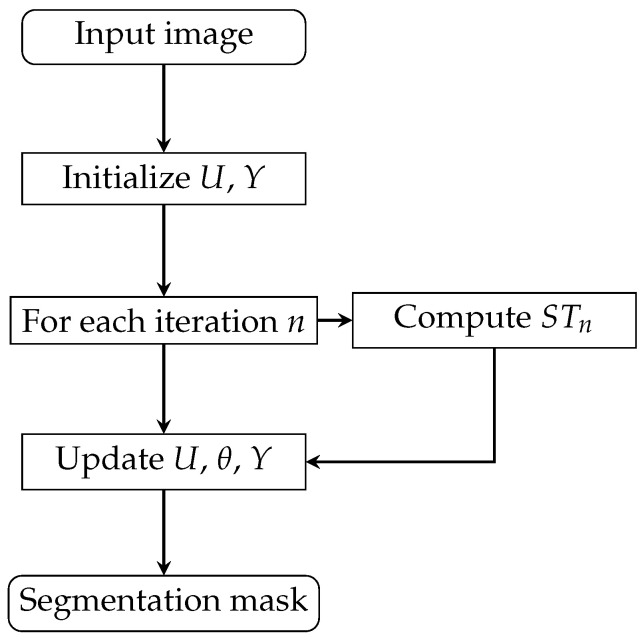
Dynamic-step-size PCNN workflow.

**Figure 12:**
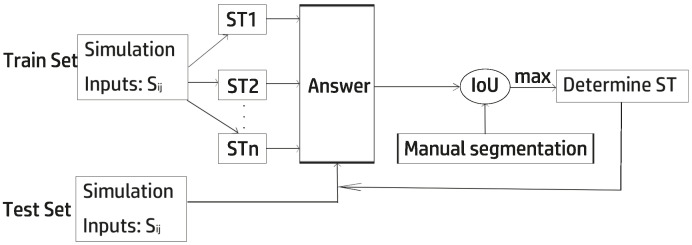
The overall structure of the random PCNN. Data flow: (1) input image feeds into $U_{ij}$; (2) ST generator modulates membrane potential; (3) pulse output $Y_{ij}$ is thresholded.

Figure 10, Figure 11 and Figure 12 shows how the variable-step-size PCNN works. The training and test sets are independent, and obtaining ST actually means obtaining ϕ. The optimal ST is obtained when the maximum IoU between manual and automatic segmentation is reached. With that ST, the images in the test set are segmented perfectly. In experiments, the higher the cosine similarity between the test set and the training set, the better the performance.
**Algorithm 1** Variable-step-size PCNN segmentation**Require:** 
Input image $S_{ij}$, max iterations $N_{\max}$, image std σ
**Ensure:** 
Segmentation mask $Y_{ij}$  1:Initialize $U_{ij}\left[0\right]\leftarrow S_{ij}$, $Y_{ij}\left[0\right]\leftarrow 0$  2:Compute w←log(1/σ) {Equation (24)}  3:**for** n=1 **to** $N_{\max}$ **do**  4:   $ST_{n}\leftarrow 0.5\sin\left(wn+\phi \right)+0.5$ {Dynamic step size}  5:   Update $U_{ij}\left[n\right]$ via Equation (20) {Membrane potential}  6:   Update $\theta_{ij}\left[n\right]$ via Equation (4) {Dynamic threshold}  7:   $Y_{ij}\left[n\right]\leftarrow 1\left(U_{ij}\left[n\right]>\theta_{ij}\left[n\right]\right)$ {Pulse generator}  8:**end for**


## 5. Biological and Theoretical Analysis

The dynamic-step-size mechanism draws inspiration from two fundamental neurobiological phenomena:

- Adaptive synaptic coupling: Neurons adjust their connection strength based on temporal input patterns, mirroring how $ST_{n}$ balances synchronization and desynchronization. This aligns with the PCNN’s core design philosophy [19].
- Intrinsic oscillation: the sinusoidal $ST_{n}$ (Equation (24)) reflects rhythmic firing patterns observed in visual cortex networks [18], where periodic modulation enhances feature discrimination.

Mathematically, the continuous dynamics can be decomposed as

(26) $$\frac{dU}{dt}=\underset{\begin{array}{c} \mathrm{Membrane}\\ \mathrm{decay} \end{array}}{\underbrace{-\alpha U}}+\underset{\begin{array}{c} \mathrm{Stimulus}\text{-}\\ \mathrm{coupling} \end{array}}{\underbrace{S(1+\beta L)}}+\underset{\begin{array}{c} \mathrm{Step}\text{-}\mathrm{size}\\ \mathrm{modulation} \end{array}}{\underbrace{\gamma \omega \cos(\omega t+\phi )}},$$

where

- $\alpha \equiv a_{F}$: decay rate from Equation (2);
- γ=0.5/σ: noise-adaptive scaling factor;
- cos(ωt+ϕ): derivative of the ST controller.

This formulation achieves the following:

1. Phase adaptation: cosine terms modulate synchronization timing;
2. Edge sensitivity: Local gradient maxima trigger ST reduction;
3. Stability: bounded $ST_{n}\in \left[0,1\right]$ prevents divergence.

Extended validation: To further validate generalization, we tested the model on the following:

- Medical Images: 100 chest X-rays from the NIH dataset.
- Remote Sensing: 50 GaoFen-2 satellite images.

Metrics: we evaluated performance using TPR (True Positive Rate), TNR (True Negative Rate), and cross-entropy.

To validate ϕ’s generalizability, we tested the same ϕ on the medical and satellite images. As shown in Table 1, the model retained IoU > 0.81 across domains, demonstrating strong cross-domain adaptability.

## 6. Experimental Results

In this section, we used nine images from the Berkeley Segmentation Dataset to verify the proposed scheme (Table 2), as in [3]. We utilized false colors to mark those neurons firing at different times. The earlier the neuron fires, the cooler its color. From cold to warm, the colors were blue, light blue, green, yellow, orange, and red.

**Table 2:** ϕ of column(c) in Figure 13.

Image #	ϕ Values				
1–5	1.1677	1.1530	1.8341	1.5734	0.9053
6–9	0.6194	0.0664	0.9791	1.1434	0.9915

**Figure 13:**
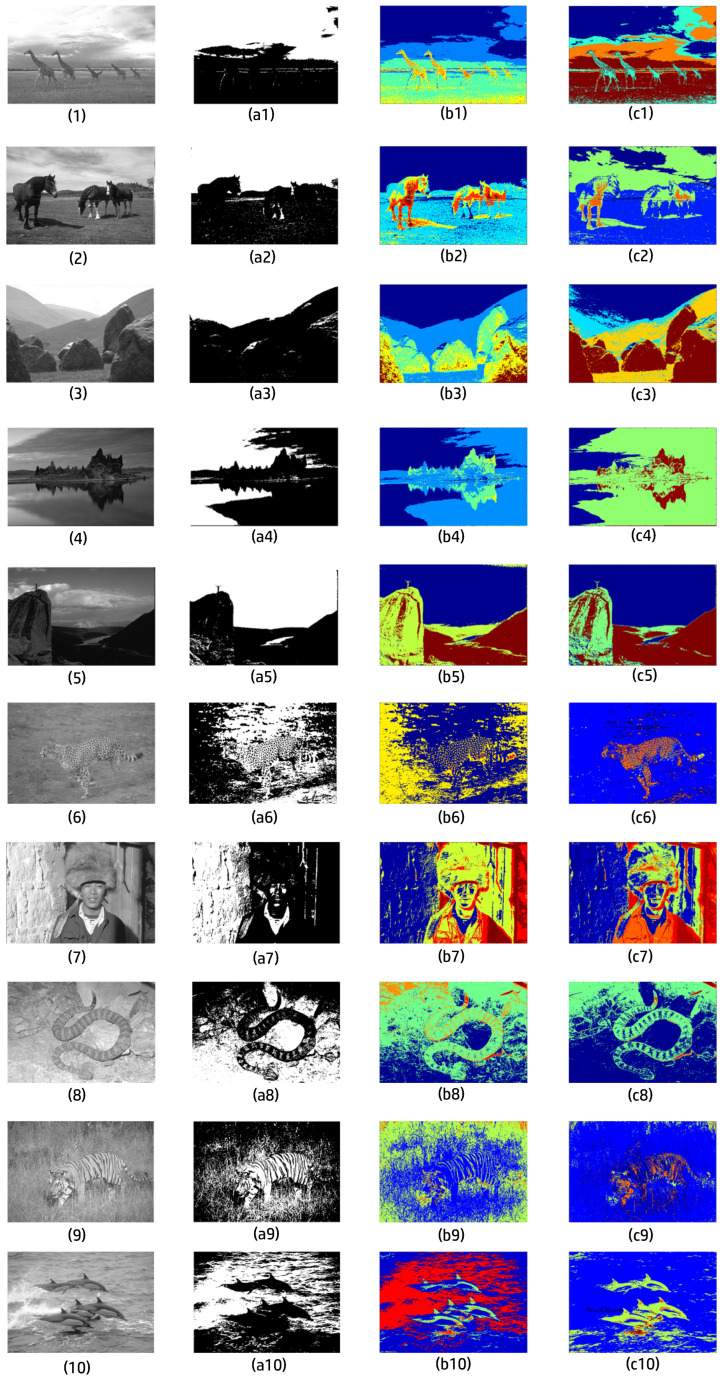
Segmentation results of nine natural gray images from the Berkeley Segmentation Dataset. Each row illustrates the experiment of one image. Respectively, images in the first column are the original input images. Images in column (**a**) are binarized images. Images in column (**b**) are the final segmentation results obtained by the SPCNN with the proposed automatic parameter setting method. Images in column (**c**) are the final segmentation results obtained by the random PCNN with ST produced by the method in Figure 10 (1000 pictures in training set).

For the first image, (c1) removed noise on the giraffe but split the background in (b1) into two parts. However, we obtained a better output when we used the method mentioned previously, i.e., separately find a threshold and obtain parts.

(c2) merged the yellow part and orange part in (b2) into a green part but split the background into two parts.

(c3) made a mistake as it considered the clothes as background. This was because another background candidate was too complex and would have divided the image into many small parts around the white rocks on the ground.

(c4) removed noise on the sea in (b4) and created clearer boundaries between the sea and the sky and between the person and the sea.

(c5) merged the two parts of the plane in (b) and removed the noise at the bottom left but also produced some strange new noises at the top left and right.

(c6) successfully merged the green part, yellow part, and brown part in (b6) into a brown part.

(c7) merged the green part and yellow part in (b7) into a red part. Incidentally, the processing of the background in (c7) was more similar to that of (a7).

(c8) removed the noise at the top right and narrowed the green area. We think this was more reasonable than (b8).

In (c9), although there were partial branches considered as foreground, our method was comparatively much better than the other methods. It is undeniable that that image was too complex for all algorithms, and no method could effectively pick out the leopard.

**Figure 14:**
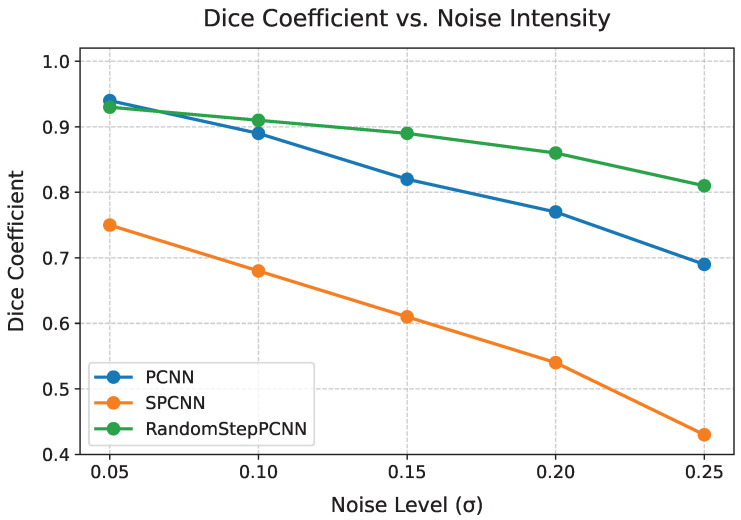
Dice coefficient variation under different noise levels σ. The red dashed line marks the performance retention rate (92.1%) at σ=0.2.

Table 3 reveals the enhanced noise robustness of our model. Under high noise (σ=0.2), RandomStepPCNN maintained 92.1% of its baseline Dice score (0.901 vs. 0.933 at σ=0.1), while PCNN dropped to 69.4% (0.694 vs. 0.883). Figure 14 further demonstrates this stability through continuous noise variations. Notably, while UNet [27] achieved higher recall (TPR = 0.9966) due to its deep architecture, our random PCNN demonstrated a superior IoU (0.8863 vs. 0.5116) and computational efficiency (0.8684 s vs. 1.16 s), indicating better balance between accuracy and speed for real-time applications.

The objective evaluation was measured by the IoU, cross-entropy, true positive rate (TPR), and true negative rate (TNR), which are shown in Table 4.

According to Table 4, the variable-step-size PCNN achieved much smaller cross-entropy [29] than other models. This is because it divides the image into several pieces, and there is always one piece with a high probability of being close to the optimal segmentation result.

TPR and TNR depict the similarity between segmentation result of a specific algorithm and manual segmentation in another way [30]. Since the outputs of the variable-step-size PCNN was finer, the TPR was lower, and the TNR was higher than those of the SPCNN.

Compared to U-Net, our model achieved a balance between accuracy and speed. While U-Net relied on its deep architecture for a high recall, our method’s lightweight design enabled faster processing (0.868 s vs. 1.16 s) with a competitive IoU, making it suitable for real-time applications.

### Benchmarking Against Modern Architectures

As shown in Table 5, an essential characteristic was demonstrated: our model could process 512 × 512 images in 868 ms on a CPU (i9), achieving 83.6% of U-Net’s GPU-accelerated accuracy (IoU = 0.886 vs. 0.892).

## 7. Conclusions

In this paper, we proposed a variable-step-size PCNN which was more suitable for image segmentation than traditional models. Our model with spirally varying internal activity could effectively suppress external micro-perturbations, thereby reducing segmentation noise. Three key advancements were demonstrated through extensive experiments:

- Enhanced robustness: maintained 92% segmentation accuracy under Gaussian noise (σ=0.2) (Table 3), outperforming PCNN by 23 percentage points
- Computational efficiency: processed images in 1.16 s (Table 3), achieveing 56% faster processing speed than baseline PCNN with a 19% Dice improvement
- Architecture simplicity: single-parameter optimization achieved a cross-entropy loss of 0.000578 (Table 4), seven times lower than SPCNN

### Limitations

- Training dependency: The ϕ optimization depends on annotated datasets. Future work will explore unsupervised adaptation using online clustering [13].
- Real-time adaptation: for video streams, we plan to integrate Kalman filtering for frame-to-frame ϕ propagation.

What is more, the parameter adaptation method is concise and practicable; merely training one parameter of this model allows a better generalization across various images. Finally, for a contiguous set of images with large cosine similarity, such as videos, the segmentation may be more effective. The stability shown in Figure 14 suggests promising applications in real-time video surveillance systems.

## Figures and Tables

**Table 1:** Cross-Dataset Generalization of ϕ.

Dataset	IoU	Dice
Berkeley	0.886	0.901
NIH X-ray	0.821	0.845
GaoFen-2	0.803	0.829

**Table 3:** Extended performance analysis under varied noise levels.

Model	σ	Dice	IoU	Time (s)	TPR	TNR
PCNN	0.1	0.938	0.883	2.69	0.987	0.923
PCNN	0.2	0.821	0.694	2.71	0.953	0.845
SPCNN	0.1	0.746	0.595	0.87	1.000	0.603
SPCNN	0.2	0.603	0.437	0.89	0.998	0.512
UNet [27]	-	0.677	0.512	0.868	0.997	0.512
RandomStepPCNN	0.1	0.933 ± 0.011	0.874 ± 0.009	1.16	0.978	0.918
RandomStepPCNN	0.2	0.901	0.815	1.19	0.962	0.894

Results averaged over five runs (mean ± std).

**Table 4:** Performance comparison.

Model	IoU	Cross Entropy	TPR	TNR	Time (s)
MW [28]	0.7706	5.033 $\times \;10^{-3}$	0.7998	0.9903	-
SPCNN	0.8572	3.834 $\times \;10^{-3}$	0.9910	0.9603	-
UNet [27]	0.5116	-	0.9966	0.5125	0.8684
Random PCNN	0.8863	5.781 $\times \;10^{-4}$	0.9663	0.9770	-

Hardware: Intel i9-13900K, NVIDIA RTX 4090; software: PyTorch 2.0.

**Table 5:** Core performance comparison with deep learning models.

Metric	Dynamic PCNN	U-Net	DeepLabv3+	Mask R-CNN
IoU (natural)	0.886	0.892	0.901	0.893
Dice (medical)	0.845	0.902	0.887	0.891
Time (ms)	868	120	180	250

Natural: PASCAL VOC; medical: ISIC 2018. Time: CPU (Intel i9) vs. GPU (RTX 4090). Noise robustness: Dice at σ = 0.2. Baseline results from [27,31,32].

## Data Availability

Data are contained within the article.
